# Preoperative intra-aortic balloon pump in patients with ST-elevation myocardial infarction undergoing urgent cardiac bypass surgery

**DOI:** 10.1007/s12471-024-01879-3

**Published:** 2024-07-03

**Authors:** Veemal V. Hemradj, Alexander J. Spanjersberg, Marit Buitenhuis, Thanasie Markou, Rik S. Hermanides, Jan-Henk Dambrink, Marcel Gosselink, Vincent Roolvink, Maarten van Leeuwen, Jan Paul Ottervanger

**Affiliations:** 1 Department of Cardiology, Isala Hospital, Zwolle, The Netherlands; 2 Department of Anaesthesiology and Intensive Care, Isala Hospital, Zwolle, The Netherlands; 3 Department of Cardiothoracic Surgery, Isala Hospital, Zwolle, The Netherlands; 4 VieCuri hospital Venlo, Venlo, The Netherlands

**Keywords:** ST-elevation myocardial infarction, Coronary artery bypass grafting, Intra-aortic balloon pump, Cardiogenic shock, Mortality, Propensity score

## Abstract

**Background:**

In patients with ST-elevation myocardial infarction (STEMI), either with or without cardiogenic shock, mechanical circulatory support with an intra-aortic balloon pump (IABP) is not associated with lower mortality. However, in STEMI patients undergoing urgent coronary artery bypass grafting (CABG), preoperative insertion of an IABP has been suggested to reduce mortality. In this study, the effect of preoperative IABP use on mortality in STEMI patients undergoing urgent CABG was investigated.

**Methods:**

All consecutive STEMI patients undergoing urgent CABG in a single centre between 2000 and 2018 were studied. The primary outcome, 30-day mortality, was compared between patients with and without a preoperative IABP. Subgroup analysis and multivariable analysis using a propensity score and inverse probability treatment weighting were performed to adjust for potential confounders.

**Results:**

A total of 246 patients were included, of whom 171 (69.5%) received a preoperative IABP (pIABP group) and 75 (30.5%) did not (non-pIABP group). In the pIABP group, more patients suffered from cardiogenic shock, persistent ischaemia and reduced left ventricular function. Unadjusted 30-day mortality was comparable between the pIABP and the non-pIABP group (13.3% vs 12.3%, *p* = 0.82). However, after correction for confounders and inverse probability treatment weighting preoperative IABP was associated with reduced 30-day mortality (relative risk 0.52, 95% confidence interval 0.30–0.88).

**Conclusion:**

In patients with STEMI undergoing urgent CABG, preoperative insertion of an IABP is associated with reduced mortality.

**Supplementary Information:**

The online version of this article (10.1007/s12471-024-01879-3) contains supplementary material, which is available to authorized users.

## What’s new?

- Use of an intra-aortic balloon pump (IABP) in ST-elevation myocardial infarction (STEMI) patients has changed since 2012.
- Mortality among STEMI patients undergoing urgent coronary artery bypass grafting (CABG) is still high.
- Crude 30-day mortality is comparable with or without preoperative IABP use in STEMI patients undergoing urgent CABG.
- After correction for propensity score, preoperative IABP use is associated with lower 30-day mortality in STEMI patients undergoing urgent CABG.

## Introduction

In patients with an ST-elevation myocardial infarction (STEMI), emergent opening of the infarct-related vessel is mandatory to improve survival [1]. In most patients, this can be achieved by primary percutaneous coronary intervention (PCI). However, in patients with complex coronary anatomy or in whom PCI is unsuccessful, urgent coronary artery bypass grafting (CABG) may be necessary. In clinical practice, an intra-aortic balloon pump (IABP) may be considered preoperatively as a ‘bridge-to-CABG’ in patients with persistent myocardial ischaemia or increased risk of haemodynamic deterioration. However, robust evidence of the benefit of this approach is lacking [2].

Previous studies regarding the effect of a preoperative IABP (pIABP) on mortality mainly focused on elective ‘high-risk’ patients, generally defined as patients with left main coronary artery disease or left ventricular ejection fraction (LVEF) < 40%, without acute myocardial infarction or cardiogenic shock (CS) [3–7]. The effect of pIABP in STEMI has been studied even less well. In a recent study, including 400 patients with non-STEMI or STEMI, in-hospital mortality was lower in pIABP patients [8]. Unfortunately, in this study, patients with CS were excluded.

In short, there is insufficient evidence regarding the effect of pIABP in patients with STEMI, either with or without CS, undergoing urgent CABG. Therefore, we performed this observational study.

## Methods

### Study design

This is an analysis of a cohort of patients hospitalised with STEMI at the Isala Heart Centre, Zwolle, the Netherlands. Our centre is a high-volume tertiary referral hospital with on-site cardiac surgery. All STEMI patients hospitalised between January 2000 and December 2018 who underwent urgent CABG were included in this study. Patients with mechanical complications (ventricular septal rupture, papillary muscle rupture), as well as type 4 or 5 myocardial infarction, were excluded to avoid bias. Data were collected in our database on coronary interventions. Primary outcome was 30-day mortality; secondary outcome was 1‑year mortality.

### Ethics

The trial was designed and conducted in accordance with the Declaration of Helsinki. The local ethics committee approved the study (reference: 210101, 04-Jan-21).

### Treatment

In all STEMI cases, emergency coronary angiography was performed. Prior to angiography, all patients received a loading dose of intravenous aspirin (500 mg), a loading dose of a P2Y12 blocker (either 600 mg clopidogrel or 180 mg ticagrelor) orally or through a nasogastric tube if intubated and intravenous unfractionated heparin (70 IU/kg).

Additional treatment with primary PCI or urgent CABG was at the discretion of the treating interventional cardiologist or heart team (consisting of an interventional cardiologist and thoracic surgeon) if indicated. The indication for IABP therapy was at the discretion of the treating interventional cardiologist. Postoperatively, all patients were admitted to the intensive care unit (ICU).

### Definitions

Urgent CABG was defined as CABG within ≤ 48 h of hospital admission. CS was defined as a systolic blood pressure ≤ 90 mm Hg for at least 30 min or the need for vasopressors to maintain a systolic blood pressure > 90 mm Hg, clinical signs of pulmonary congestion and end-organ hypoperfusion (cool extremities, altered mental status, or a urine output of < 30 ml/h [9]). Shock index (SI) was defined as the quotient of heart rate and systolic blood pressure at admission and was considered elevated when ≥ 0.7 [10]. Persistent ischaemia (PI) was defined as persisting angina pectoris or a thrombolysis in myocardial infarction (TIMI) score < 3 post-PCI or when PCI was not performed. Patients who received an IABP before CABG (prior to sternotomy) were defined as the pIABP group. Patients who did not receive an IABP or received an IABP after sternotomy were defined as the non-pIABP group.

In 2012, the IABP-SHOCK II trial results were published, demonstrating that IABP use in STEMI complicated by CS did not reduce mortality [11]. After this publication, IABP use possibly declined. Therefore, we used the ‘after year 2012’ parameter in our analysis.

At least 365 days after admission, the municipal registry was checked to see whether all patients were still alive. Thirty-day all-cause mortality was defined as mortality within 30 days of admission, and 1‑year all-cause mortality within 365 days of admission.

### Statistics

Normally distributed continuous variables are reported as mean ± standard deviation (SD) and compared with ANOVA corrected for multiple testing by the Bonferroni method. Skewed distributed variables are presented as median and compared using the Wilcoxon rank-sum test. Categorical variables are presented as proportions and compared by a chi-square test. Kaplan Meier analyses were calculated, and a log-rank test was used to compare survival between the pIABP and non-pIABP group. Subgroup analysis of 30-day mortality, corrected for age, is presented in a Forrest plot (Fig. S1, Electronic Supplementary Material).

Discarding patients with missing data may lead to a loss of precision and biased results. Therefore, missing data were imputed in SPSS using five imputed datasets. These sets were analysed and then the results were pooled.

Retrospective studies are prone to bias. Confounders were defined as covariates associated with both treatment group and outcome. Inverse probability treatment weighting (IPTW) was applied to compensate for imbalance in these covariates [12]. A propensity score was calculated from age, gender, comorbidity, systolic blood pressure on admission, LVEF, whether a PCI was performed and the result thereof (TIMI flow < 3), CABG after 24 h and admission period (before or after 2012), with the appropriate interaction terms. Although EuroSCORE is derived from preoperative clinical parameters, including IABP, we did not use this value for our propensity score. A receiver operating characteristic (ROC) analysis with calculation of the C‑statistic was performed to test if this propensity score was an adequate predictor, with a value of > 0.7 indicating a good model. Imbalance in covariates was expressed as standardised mean difference (SMD) before and after IPTW. Multivariable analysis was performed using an IPTW linear regression model for 30-day mortality. Analyses were performed with SPSS version 28.0 (IBM Corp., Armonk, NY, USA) and R statistical software (version 3.6.2).

## Results

A total of 246 patients were included, with 171 patients (69.5%) in the pIABP group and 75 patients (30.5%) in the non-pIABP group. Baseline characteristics are summarised in Tab. 1, with imbalance expressed as SMD before and after IPTW. Age, gender and cardiovascular risk factors were comparable between the two groups. In the pIABP group, systolic blood pressure at hospitalisation was lower (median 120 mm Hg vs 130 mm Hg, *p* < 0.01); LVEF < 30% and PI were more prevalent (25.1% vs 13.3%, *p* < 0.01, and 50.6% vs 30.7%, *p* < 0.01, respectively). Furthermore, CABG after 24 h was more frequent in the non-pIABP group. Finally, whereas pIABP is a significant risk factor, the median logistic EuroSCORE was higher in the pIABP group (20.0 vs 8.0, *p* < 0.01).

**Table 1:** Baseline characteristics of ST-elevation myocardial infarction patients undergoing urgent coronary artery bypass grafting (*CABG*) with versus without a preoperative intra-aortic balloon pump (*IABP*)

		Original data				Inverse probability treatment weigthed		
		IABP pre-CABG		SMD	*p*-value	IABP pre-CABG		SMD
		No *n* = 75	Yes *n* = 171			No *n* = 226	Yes *n* = 224	
Age at CABG		67 (59–75)	66 (58–75)	0.037	0.823	66 (59–75)	66 (58–74)	−0.010
Female		19 (25.3%)	42 (24.6%)	−0.018	0.897	67 (29.8%)	59 (26.4%)	−0.074
*Risk factors*								
Diabetes mellitus		13 (17.3%)	21 (12.4%)	0.146	0.280	34 (14.9%)	30 (13.3%)	0.045
Hypertension		37 (50.0%)	72 (42.4%)	0.154	0.269	96 (42.8%)	103 (45.8%)	−0.059
Smoking		19 (25.7%)	65 (38.5%)	−0.270	0.054	53 (23.2%)	79 (35.4%)	−0.270
COPD		5 (6.7%)	12 (7.0%)	−0.014	0.920	19 (8.4%)	15 (6.8%)	0.062
Extracardiac arteriopathy		5 (6.7%)	11 (6.4%)	0.009	0.945	21 (9.2%)	14 (6.4%)	0.106
Log EuroSCORE		8.0 (4.0–18.8)	20.0 (11.0–37.2)	−0.751	< 0.001	15.0 (5.0–21.0)	23.7 (10.4–35.0)	−0.546
*History*								
Prior MI		9 (12.2%)	13 (7.6%)	0.157	0.258	19 (8.4%)	17 (7.6%)	0.029
Prior PCI		7 (9.3%)	11 (6.5%)	0.109	0.429	22 (9.5%)	22 (10%)	−0.014
Prior CVA		4 (5.4%)	6 (3.5%)	0.094	0.497	9 (4.0%)	8 (3.4%)	0.033
*Clinical characteristics on admission*								
After 2012		34 (45.3%)	36 (21.1%)	0.553	< 0.01	69 (30.3%)	64 (28.7%)	0.036
Systolic blood pressure, mm Hg		130 (112–150)	120 (100–140)	0.305	0.007	124 (110–140)	125 (104–141)	−0.009
Heart rate		80 (69–91)	78 (70–95)	−0.056	0.905	82 (69–96)	82 (70–95%)	−0.029
Shock index		0.60 (0.50–0.75)	0.64 (0.53–0.83)	−0.206	0.091	0.70 (0.52–0.78)	0.70 (0.53–0.82)	0.012
Shock index > 0.7		22 (30.6%)	60 (40.8%)	−0.212	0.141	84 (38.3%)	79 (36.8%)	0.030
Cardiogenic shock		11 (14.7%)	43 (25.1%)	0.056	0.068	46 (20.2%)	45 (20.2%)	−0.002
Cardiac arrest		13 (17.3%)	31 (19.1%)	0.210	0.199	48 (21.0%)	39 (17.3%)	0.094
CABG after 24 h		40 (53.3%)	67 (39.2%)	0.287	0.039	108 (47.7%)	100 (44.7%)	0.061
LVEF	> 50%	28 (37.3%)	30 (17.5%)	−0.480	0.002	54 (23.9%)	55 (24.4%)	0.000
	30–50%	37 (49.3%)	98 (57.3%)			126 (55.5%)	122 (54.5%)	
	< 30%	10 (13.3%)	43 (25.1%)			47 (20.6%)	47 (21.1%)	
*Laboratory data on admission*								
Haemoglobin		8.8 (8.1–9.4)	8.8 (8.2–9.3)	−0.112	0.825	8.6 (8.0–9.4)	8.7 (8.1–9.3)	−0.115
Glucose		8.9 (7.4–12.0)	8.8 (7.4–11.5)	−0.044	0.824	10.2 (7.4–12.0)	9.9 (7.4–11.0)	0.066
Creatinine		86 (75–97)	87 (71–104)	−0.066	0.813	92 (75–107)	90 (71–100)	0.041
*Angiographic data*								
LM involved		31 (41.3%)	85 (49.7%)	−0.168	0.226	97 (42.8%)	113 (50.6%)	−0.157
LAD involved		66 (88.0%)	146 (85.4%)	0.076	0.584	196 (86.5%)	192 (85.6%)	0.026
Cx involved		53 (70.7%)	130 (76.0%)	−0.122	0.376	165 (73.1%)	177 (78.8%)	−0.135
RCA involved		53 (70.7%)	127 (74.3%)	−0.081	0.557	147 (65.1%)	161 (72.1%)	−0.151
Multivessel disease		69 (92.0%)	155 (90.6%)	0.047	0.731	209 (92.2%)	208 (92.7%)	−0.021
PCI performed		46 (61.3%)	84 (49.1%)	0.245	0.077	121 (53.4%)	119 (52.9%)	0.010
Stent placed		13 (17.3%)	15 (8.8%)	0.271	0.052	39 (17.2%)	19 (8.5%)	0.307
TIMI-flow post PCI < 3		23 (30.7%)	86 (50.6%)	−0.406	0.004	98 (43.4%)	96 (43.0%)	0.008

*COPD* chronic obstructive pulmonary disease, *CVA* cerebral vascular accident, *Cx* circumflex coronary artery, *LAD* left anterior descending coronary artery, *LVEF* left ventricular ejection fraction, *LM* left main coronary artery, *MI* myocardial infarction, *PCI* percutaneous coronary intervention, *RCA* right coronary artery, *SMD* standardised mean difference, *TIMI* thrombolysis in myocardial infarction

Table S1 (Electronic Supplementary Material) presents a comparison of those patients who survived the first 30 days and those who did not. Patients who died within 30 days were significantly older (median 74 vs 65 years, *p* < 0.01) and more often female (41.9% vs 22.3%, *p* < 0.01). Furthermore, SI ≥ 0.7, CS at admission, cardiac arrest and LVEF < 30% were significantly more prevalent in patients who died within 30 days (69.2% vs 33.2%, *p* < 0.01; 51.6% vs 17.7%, *p* < 0.01; 45.2% vs 24.0%, *p* < 0.01; 74.2% vs 14.0%, *p* < 0.01, respectively). CABG after 24 h was more prevalent in patients surviving more than 30 days. Median blood glucose and creatinine were significantly higher in patients who did not survive beyond 30 days (10.9 vs 8.7 mmol/l, *p* = 0.29 and 104 vs 85 mmol/l, *p* < 0.01, respectively).

Tab. 2 gives an overview of the clinical course of patients in both groups. Extracorporeal bypass time and ICU stay were significantly longer in pIABP patients (median 123 min versus 94 min and 50 h versus 27 h, respectively).

**Table 2:** Clinical course and outcome of ST-elevation myocardial infarction patients undergoing urgent coronary artery bypass grafting (*CABG*) with versus without preoperative intra-aortic balloon pump (*IABP*)

	No IABP (*n* = 75)	pIABP (*n* = 171)	*p*
*Clinical course*			
IABP per-/post-CABG (%)	17.3	100.0	< 0.01
ECMO per-/post-CABG (%)	4.0	4.7	0.39
LVAD per-/post-CABG (%)	1.3	4.1	0.45
Off-pump procedure (%)	4.0	4.1	0.97
ECC time (min)	94 (78–124)	123 (91–183)	< 0.01
Aorta occlusion time (min)	54 (45–63)	59 (44–72)	0.31
Intraoperative blood loss (ml)	325 (250–600)	350 (200–630)	0.85
*Postoperative course*			
ICU stay (h)	27 (20–81)	50 (25–118)	0.03
Ventilator time ICU (h)	7 (4–17)	13 (7–40)	0.09
Creatinine max. (µmol/l)	86 (69–140)	93 (70–130)	0.16
Blood loss ICU (ml)	593 (365–1110)	785 (465–1300)	0.08
CRRT (%)	4.0	1.8	0.29
Transfusion RBC (units first 72 h)	3 (2–5)	2 (1–5)	0.18
Resternotomy (%)	6.7	7.0	0.92
Outcome			
– Mortality 30 days (%)	13.3	12.3	0.82
– Mortality 1 year (%)	14.7	14.0	0.90

*CRRT* continuous renal replacement therapy, *ECC* extracorporeal circulation, *ECMO* extracorporeal membrane oxygenation, *IABP* intra-aortic balloon pump, *ICU* intensive care unit, *LVAD* left ventricular assist device, *RBC* red blood cells

From the ROC analysis of study group by propensity score, a C-statistic was calculated of 0.78 (95% confidence interval [CI] 0.72–0.84), indicating a good model.

### Outcome

**Fig. 1:**
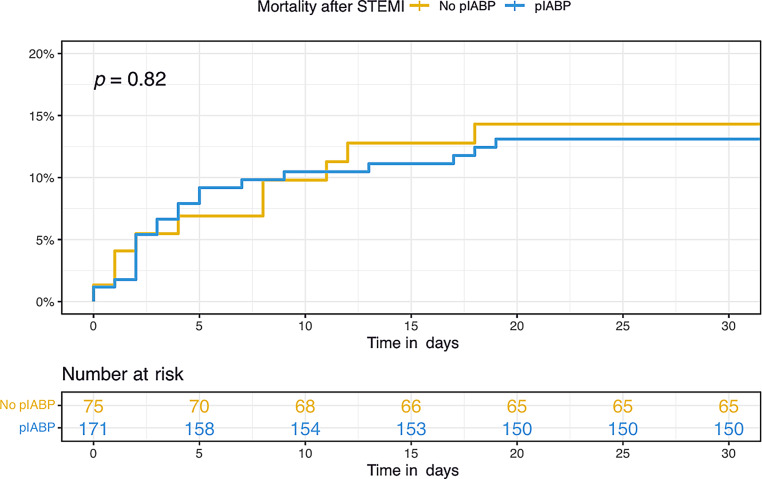
Kaplan-Meier analysis of 30-day all-cause mortality. (*pIABP* preoperative intra-aortic balloon pump, *STEMI* ST-elevation myocardial infarction)

Crude 30-day all-cause mortality was 12.6% and was not statistically different between the pIABP and non-pIABP group (12.3% vs 13.3%, *p* = 0.82); Tab. 2; Fig. 1. One-year all-cause mortality was 14.2% and not statistically different between the two groups (14.0% vs 14.7%, *p* = 0.90); Tab. 2; Fig. 1.

After IPTW, the imbalance in covariates associated with 30-day mortality was less than 0.1. A linear regression model demonstrated a mortality benefit in the pIABP group, a relative risk (RR) of 0.52 (95% CI 0.30–0.88) for 30-day mortality and of 0.57 (95% CI 0.43–0.96) for 1‑year mortality.

Subgroup analysis did not show subgroups that could specifically benefit from pIABP (Fig. S1, Electronic Supplementary Material).

## Discussion

This is the first study to investigate the effect of pIABP in STEMI patients undergoing urgent CABG, including those with CS and cardiac arrest. The key findings of this study were that patients undergoing urgent CABG with pIABP had (1) still high 30-day and 1‑year mortality, (2) similar 30-day and 1‑year mortality to the non-pIABP group despite being at higher risk at baseline, (3) similar postoperative complications to those without pIABP, (4) no particular subgroups with a significantly different treatment effect.

Despite the significant differences in clinical presentation at baseline in favour of the non-pIABP group, both groups had similar unadjusted 30-day and 1‑year mortality. However, after adjusting for relevant confounders such as age, gender, comorbidity, CS, poor LVEF and PI, using IPTW, patients in the pIABP group had a significant survival benefit.

It has previously been demonstrated that an IABP increases coronary blood flow, decreases the workload of the myocardium and increases the cardiac output by 0.5–1 l/min [13, 14]. The rationale of pIABP is that these haemodynamic improvements can precondition the ischaemic heart so that the risk of postoperative low-cardiac output syndrome, postoperative CS and mortality is reduced. There is conflicting evidence regarding pIABP in elective patients. In the first randomised clinical trials (RCTs) by Christenson et al., pIABP use reduced in-hospital mortality in high-risk patients, generally defined as patients with left main disease or LVEF < 40% undergoing elective CABG [3–5]. A meta-analysis of 10 RCTs on pIABP in high-risk surgery patients also suggests a mortality benefit [15].

However, in two more recent RCTs with larger study populations, pIABP use did not improve outcome in high-risk cardiac bypass surgery patients [7, 16]. The primary outcome in these studies was a composite endpoint of major morbidity and mortality. Rocha Ferreira et al. suggest that in the case of optimal preoperative treatment, an IABP has little additive value [16]. These results are in line with the findings of the BCIS-1 trial, demonstrating that IABP insertion prior to complex PCI does not reduce mortality [17]. Thus, the value of pIABP in elective patients is still questionable.

In patients with STEMI, whether complicated by CS or not, the balance between myocardial oxygen demand and supply is even more disturbed than in elective high-risk patients. Therefore, haemodynamic support in these patients could be of more importance.

In a previous study in patients with non-STEMI and STEMI, the authors concluded that pIABP was associated with reduced in-hospital mortality [8]. Patients were included in this study if CABG was performed within 5 days of admission. Time to surgery is not reported; however, this time interval may have increased selection bias. Patients with persisting complaints or deteriorating haemodynamics were likely to have had surgery sooner than relatively stable patients who could wait until day 5. To avoid this risk of selection bias, we included only patients who underwent CABG within 2 days in this study and used the factor ‘CABG after 24 h’ in our analysis and propensity score. Furthermore, the authors excluded CS patients based on the IABP SHOCK-II trial findings. In this landmark trial, IABP use did not reduce mortality in STEMI complicated by CS [11]. However, in this study, most patients were treated with primary PCI, with only 3.5% undergoing immediate CABG. Therefore, the results of this study cannot be extrapolated to patients with STEMI and CS undergoing urgent CABG. In our study, all patients with STEMI, as well as those with CS and cardiac arrest, were included.

In patients with persisting or ongoing ischaemia, an IABP can be inserted as a bridge-to-CABG, although robust evidence is lacking. Unsuccessful PCI in STEMI is associated with higher mortality than STEMI with successful PCI, especially in patients with CS [18, 19]. In previous retrospective studies, IABP use in patients with PI after primary PCI was associated with reduced mortality [19, 20]. However, more recently, IABP insertion did not reduce mortality in patients with large anterior STEMI and PI [21]. In our study, subgroup analysis did not demonstrate reduced mortality in patients with PI treated with a pIABP.

A possible explanation for previous failure to demonstrate the benefit of a pIABP could be an increased complication risk counterbalancing the positive effect of IABP use. An IABP is inserted via the femoral artery through a 7 French sheath and is associated with an increased bleeding risk and higher mortality [17]. Adequate positioning, just below the aortic arch on a chest X‑ray, is imperative for good functioning, whereas malpositioning is associated with a 4- to 13-fold increase in life-threatening complications [22]. Furthermore, thromboembolic or ischaemic events may occur due to blocking major abdominal vessels or dislodging of atheromatous debris from the aortic vessel wall [23]. Unfortunately, our study did not have sufficient data to investigate these complications.

As an IABP can improve cardiac output, coronary flow and afterload, we found an improved clinical outcome in our study. Perhaps a device generating more haemodynamic support, such as Impella (Abiomed, Danvers, MA, USA), could further improve the clinical outcome. The Impella is a catheter-based microaxial flow pump placed across the aortic valve into the left ventricle (LV). It unloads the LV and increases the cardiac output and coronary blood flow [24–26]. It also increases the cardiac power output, which was previously shown to be the strongest haemodynamic predictor of mortality in patients with STEMI complicated by CS [27, 28]. Impella use in elective high-risk PCI does not reduce mortality compared to an IABP but does improve procedural success [29]. However, there are only limited data on Impella use in high-risk CABG, moreover related only to postoperative Impella use [30]. Until now, there has been no study on the effect of preoperative Impella use in urgent CABG.

### Limitations

There are several limitations to this study. First, this is a single-centre study with an observational design. Second, the small sample size, especially the non-pIABP group, limits subgroup analysis. Third, implantation of a pIABP was not dictated by a protocol, increasing the potential for selection bias. We partially tried to overcome this by performing a propensity score analysis. Finally, we did not have data on several variables, such as lactate and haemodynamic parameters during hospital stay. Given the above-mentioned limitations, the findings of our study should be interpreted as hypothesis generating.

## Conclusion

In conclusion, our findings support routine pIABP use in STEMI patients with an indication for urgent CABG, in whom mortality is shown to be reduced. However, an adequately powered RCT is needed to reach final conclusions for patients with CS undergoing urgent CABG. Also, further research is necessary to investigate the effect of other haemodynamic assist devices in STEMI patients undergoing urgent CABG.

### Supplementary Information

**Fig. S1** Subgroup analysis of 30-day mortality, corrected for age, in a Forrest plot

**Table S1** STEMI patients undergoing urgent CABG who died within 30 days versus those who survived 30 days

## Data Availability

The data used for this manuscript can be obtained from the corresponding author upon request.
