# Supplementary material for: Preoperative intra-aortic balloon pump in patients with ST-elevation myocardial infarction undergoing urgent cardiac bypass surgery
Source: Neth Heart J. 2024 Jul 3;32(7-8):276–82. doi: 10.1007/s12471-024-01879-3 (PMC11239630; doi:10.1007/s12471-024-01879-3)
Supplement: Supplementary file 1 — Fig. S1 Subgroup analysis of 30-day mortality, corrected for age, in a Forrest plot [file 12471_2024_1879_MOESM1_ESM.docx]

**Fig. S1** Subgroup analysis of 30-day mortality, corrected for age, in a Forrest plot


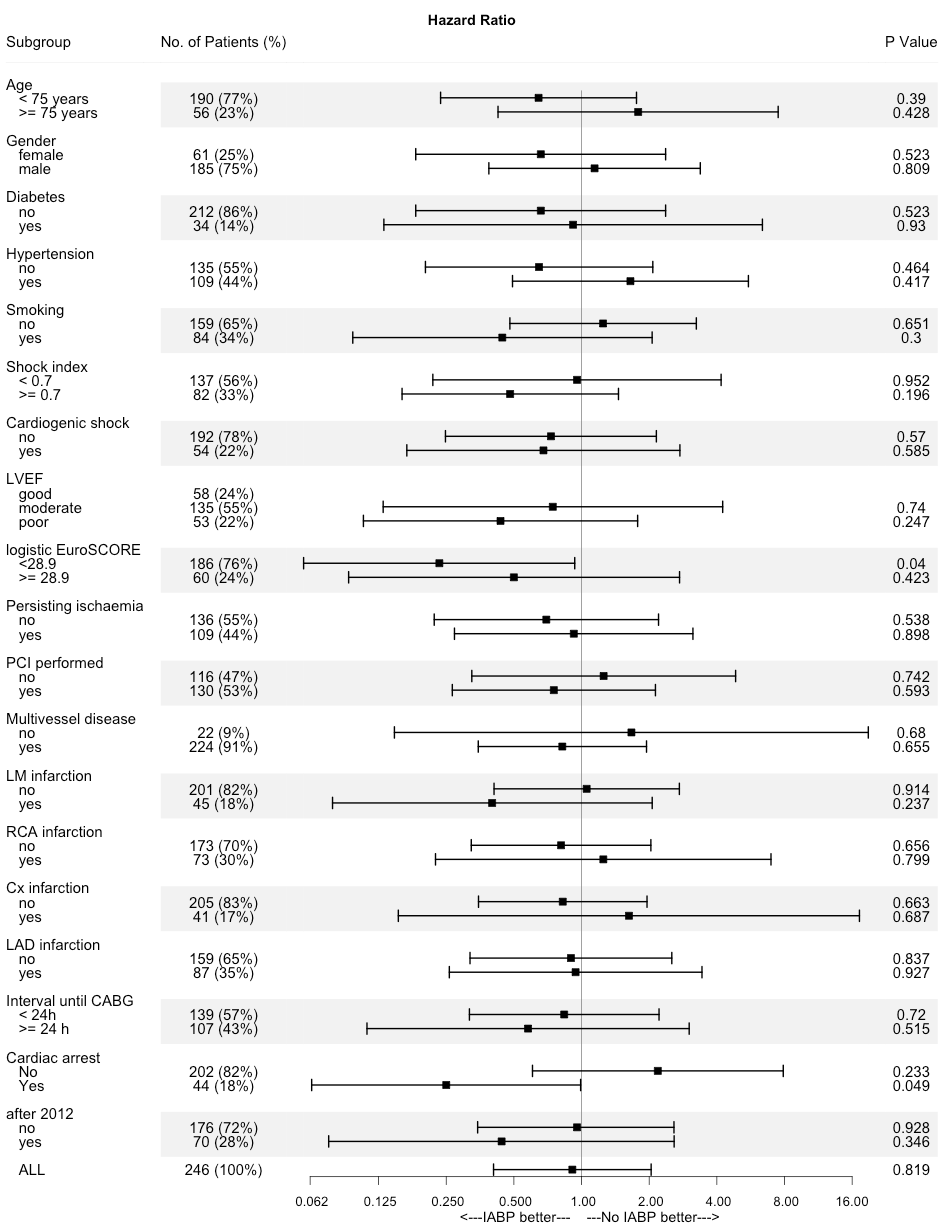


Abbreviations: LVEF – left ventricular ejection fraction, PCI – percutaneous coronary intervention, LM – left main, RCA - right coronary artery, Cx – circumflex , LAD – left anterior descending.
