# Supplementary material for: Preoperative intra-aortic balloon pump in patients with ST-elevation myocardial infarction undergoing urgent cardiac bypass surgery
Source: Neth Heart J. 2024 Jul 3;32(7-8):276–82. doi: 10.1007/s12471-024-01879-3 (PMC11239630; doi:10.1007/s12471-024-01879-3)
Supplement: Supplementary file 2 — Table S1 STEMI patients undergoing urgent CABG who died within 30 days versus those who survived 30 days [file 12471_2024_1879_MOESM2_ESM.docx]

**Table S1** STEMI patients undergoing urgent CABG who died within 30 days versus those who survived 30 days

|  | | Mortality at 30-days  NO  N = 215 | YES  N = 31 |  | p-value |
| --- | --- | --- | --- | --- | --- |
| *Age at CABG* | | 65 (57-74) | 74 (65-78) |  | .002 |
| *Female* | | 48 (22.3%) | 13 (41.9%) |  | .018 |
| Risk Factors | |  |  |  |  |
| *Diabetes Mellitus* | | 29 (13.6%) | 5 (16.7%) |  | .644 |
| *Hypertension* | | 93 (43.5%) | 16 (53.3%) |  | .308 |
| *Smoking* | | 76 (35.8%) | 8 (25.8%) |  | .272 |
| *COPD* | | 14 (6.5%) | 3 (9.7%) |  | .516 |
| *Extracardiac arteriopathy* | | 15 (7.0%) | 1 (3.2%) |  | .429 |
| *Log EuroSCORE* | | 14.1 (7.0-24.9) | 40.1 (24.4-55.8) |  | <.001 |
| History | |  |  |  |  |
| *Prior MI* | | 18 (8.5%) | 4 (12.9%) |  | .419 |
| *Prior PCI* | | 16 (7.5%) | 2 (6.5%) |  | .838 |
| *Prior CVA* | | 8 (3.8%) | 2 (6.5%) |  | .479 |
| Clinical characteristics on admission | |  |  |  |  |
| *After year 2012* | | 64 (29.8%) | 6 (19.4%) |  | .230 |
| *Systolic blood pressure* | | 125 (110-145) | 110 (85-126) |  | .005 |
| *Heart rate* | | 78 (70-91) | 85 (70-110) |  | .082 |
| *Shock index* | | .62 (.52-.77) | .86 (.59-1.00) |  | <.001 |
| *Shock index > 0.7* | | 64 (33.2%) | 18 (69.2%) |  | <.001 |
| *Cardiogenic shock* | | 38 (17.7%) | 16 (51.6%) |  | <.001 |
| *Cardiac arrest* | | 30 (24.0%) | 14 (45.2%) |  | <.001 |
| *CABG after 24h* | | 101 (47%) | 6 (19.4%) |  | .004 |
| *LVEF* | *> 50%* | 56 (26.0%) | 2 (6.5%) |  | <.001 |
| *30-50%* | | 129 (60.0%) | 6 (19.4%) |  |  |
| *< 30%* | | 30 (14.0%) | 23 (74.2%) |  |  |
| Laboratory data on admission | |  |  |  |  |
| *Haemoglobin* | | 8.8 (8.2-9.4) | 8.5 (7.8-9.0) |  | .100 |
| *Glucose* | | 8.7 (7.3-11.3) | 10.9 (7.8-14.0) |  | .029 |
| *Creatinine* | | 85 (71-97) | 104 (89-118) |  | <.001 |
| Angiographic data | |  |  |  |  |
| *LM involved* | | 99 (46.0%) | 17 (54.8%) |  | .359 |
| *LAD involved* | | 188 (87.4%) | 24 (77.4%) |  | .131 |
| *Cx involved* | | 162 (75.3%) | 21 (67.7%) |  | .364 |
| *RCA involved* | | 158 (73.5%) | 22 (71.0%) |  | .767 |
| *Multivessel disease* | | 198 (92.1%) | 26 (83.9%) |  | .134 |
| *PCI performed* | | 113 (52.6%) | 17 (54.8%) |  | .812 |
| *Stent placed* | | 23 (10.7%) | 5 (16.1%) |  | .373 |
| *TIMI-flow post PCI <3* | | 91 (42.5%) | 18 (58.1%) |  | .104 |

Abbreviations:

CABG – coronary artery bypass grafting

COPD – chronic obstructive pulmonary disease

CVA – cerebral vascular accident

Cx – circumflex coronary artery

LAD – left anterior descending coronary artery

LVEF – left ventricular ejection fraction

LM – left main coronary artery

MI – myocardial infarction

PCI – percutaneous coronary intervention

RCA – right coronary artery

TIMI - Thrombolysis in Myocardial Infarction
